# Profession Loss Crisis at an Old Age: Specific Features, Factors, and Mechanisms of Coping

**DOI:** 10.3390/bs9120152

**Published:** 2019-12-11

**Authors:** Elvira Symanyuk, Georgy Borisov, Daria Berdnikova, Olga Tomberg, Alexandra Ryabukhina

**Affiliations:** 1 Department of General and Social Psychology, Ural Federal University, Yekaterinburg 620083, Russia; georgy.borisov@urfu.ru (G.B.); dariyab@bk.ru (D.B.); ryabuchina@mail.ru (A.R.); 2 Department of Foreign Languages and Translation, Ural Federal University, Yekaterinburg 620083, Russia; o.v.tomberg@urfu.ru

**Keywords:** professional development, old age, professional crises, profession loss crisis, psychobiographic method, teachers, activity, factors that initiate profession loss crisis, mechanisms of coping with crisis

## Abstract

This article discusses the specific characteristics of profession loss crisis at an old age. Profession loss crisis is the last normative crisis of personal professional development that is caused by the completion of one’s professional biography after reaching a certain age. The research employs a psychobiographic method and a critical events method. These methods are based on the use of a formalized biographical questionnaire worked out by Norakidze V.G. and reconstructed by Zeer E.F. The authors have identified and provided a detailed description of the main factors that cause profession loss crisis: random events, adverse circumstances while implementing professional plans, etc. The article outlines the main strategies for coping with this crisis: changing jobs, re-training, the assistance of colleagues and administration, etc. The authors suggest technologies to minimize the effects of these factors and overcome profession loss crisis effectively.

## 1. Introduction

Current developments in social and economic spheres, and, in particular, increasing retirement ages in many countries, have heightened the need for research into the psychology of people of “the third age”. A conceptual focus in this research is placed on analyzing characteristic features of professional development at an old age, finding factors that contribute to effective professional activity at this age, working out technologies to cope with a profession loss crisis, and acquiring a new social role. 

To date, the problem of profession loss crisis has received scant attention in the research literature, though it has proven to be a topical social issue. Previous sociological research has established that after finishing their professional career and retiring many people consider it difficult to find a social niche. It leads to a significant drop in a living standard and a loss of social ties with peers and other age groups. 

Within this perspective, this article aims to find the specific features of profession loss crisis at an old age.

The following research objectives would facilitate the achievement of this aim:

1) Analyzing approaches to the concept of profession loss crisis and characteristic features that help to cope with it.
2) Conducting an empirical study in order to (a) define the factors that predetermine profession loss crisis; (b) find the changes in a person’s activity while undergoing the crisis; (c) suggest ways to cope with the crisis.
3) Investigating structural changes of the actor in the process of profession loss crisis and ways to cope with it.

There are several approaches to the concepts of retirement and profession loss crises. Some scholars consider the retirement crisis a myth, since it was discovered that people’s mental, physiological, and social conditions remain safe. 

A study conducted by Spanish researchers suggests that the concept of the retirement crisis is not so topical today. The world recession as noted by Fuenta V.S. [1] leads to “natural selection” in the labor market—a situation in which the most adapted people remain in their workplaces. Therefore, older employees with substantial professional experience are much sought-after by various employers. These employees have a longer professional life cycle and this trend has a positive social effect because professional injuries are reduced in this case (due to the employees’ professional experience). However, other researchers [2,3] are not so optimistic about the situation and argue that the global recession is a reason for permanent stress for the working population. This stress may cause serious diseases that can force a person to leave their profession early. Thus, the concept of the retirement crisis is becoming a topical issue. It is necessary to identify the factors that could be helpful to cope with it.

The most important among these factors are health, social and economic status, and structure of personality [4]. Another study on this topic shows that retirement has both advantages (free time) and disadvantages (worsening of financial situation, change in social status), but, in general, it allows a person to maintain well-being. Health conditions, social ties, and an optimistic worldview can be considered as factors that alleviate the process of retirement [5]. In addition, the retirement crisis is overcome more easily if a person does not have a strong identification with their profession and if this profession is not an aim within itself for the person [6].

Christina R. Victor considers the retirement crisis as an intermediate state and singles out five stages within it: increasing interest as retirement approaches, initial euphoria, slight stress, adjustment to a new lifestyle, and peace of mind [7].

Research shows that the process of retiring is accompanied by a decrease in cognitive functions [8] and symptoms of depression [9].

In addition, the retirement crisis is complicated by a combination of factors that place high demands on the psychological resources of the person: a threat of ego identity, painful awareness of the finitude of being, a need to reassess goals, activities, and roles [10].

Retirement is also associated with such psychological phenomena as identity disorder, indecision, decrease in self-confidence, feeling of emptiness, search for new ways to participate in public life, reassessment of one’s own life, aging experience, fear of death, high need for care of others, and self-actualization. To overcome the retirement crisis successfully it is advisable to find new interests and leisure activities [11].

The factors that provide successful end of one’s professional activity include a high degree of needs satisfaction, love of one’s job, and retirement planning [12,13].

Profession loss crisis is considered to be one of the normative crises of professional development. Crises of individual professional development are defined as short periods of radical shifts in professional conscience that are accompanied by a change in the direction of professional development. The main characteristic features of professional crises are losing a feeling of novelty, being out of touch, decreasing a professional level, inner discomfort, a need for self-reassessment, self-depreciation, fatigue, and a feeling that one’s resources are limited. 

Profession loss crisis is the last normative crisis of professional development. This crisis is caused by the end of a person’s professional career when he/she retires. Many employees face a crisis in the pre-retirement period; it is connected with mastering a new social role and behavior patterns and making plans for the future. Retirement is inevitably accompanied by a diminishing social and professional field, as well as a loss of financial resources. The severity of the crisis depends on the character of the professional activity (manual labor workers undergo this process more smoothly), marital, and health status.

L. S. Vygotsky argued that a peculiar feature of the crisis is a blurring of the boundaries separating the beginning and end of the crisis from adjacent ages. However, in the midst of the crisis there comes a “climax when the crisis reaches its peak” [14]. He proposes considering this climax as a starting point with approximately half a year from each side of it (towards the beginning and the end of the crisis). In this respect Vygotsky L.S. proposes three phases of crisis development:

- Pre-critical; at this stage a predominant role is played by factors that trigger an increase in mental tension and dissatisfaction;
- Critical; when structural changes in the personality occur, a system of motives is changed and values are re-assessed;
- Post-critical; the process of overcoming the crisis.

Coping with the crisis can be twofold: it may be either the restoration of the life interrupted by the crisis, its rebirth, or its transformation into a new life as such. Any of these cases result in life construction and self-creation.

Successful crisis resolution stimulates the inflow of a person’s strength, a desire to master new spheres of reality, increases their motivation for achievements, and contributes to the development of new abilities.

Factors that trigger crises of teachers’ professional development are investigated by Antsyferova L.I. [15], Zeer E.F. [16], Klimova E.A. [17], Kuzmina N.V. [18], and Markova A.K. [19]. These factors can be divided into subjective and objective groups. 

The group of objective factors comprises a change in the main activity or the way of doing it, a change in a social development situation, age-related psychological and psychophysiological changes, professional deformation and stagnation, deterioration or improvement of the social and economic situation, and fortuitous or unfavorable events. 

The second group of factors is caused by subjective personality qualities: inner conditions of personality development and the person’s activity that is necessary for self-development. 

Personal activity is crucial for personality development. The subjective activity of a person is shaped by a system of dominating needs, motives, interests, orientations, etc. Much of the literature on the topic emphasizes the fact that the essential features of the definition “activity” refer to quantitative and qualitative characteristics of the intensity level of a process or interaction, the potential of a person’s abilities, and a source of any process or interaction [20,21]. The research literature on humanistic and existential psychology argues that the main source of activity is one’s striving for self-actualization and meaning, as well as being an actor of one’s own life and bearing responsibility for it. Frankl V. provides examples that intentional activity, i.e., a type of activity that is directed at something outside oneself, helps a person to acquire new meanings in life at an old age, including the period of completing a professional career [22]. Johnson S.J. [23] suggests that a person’s ability to direct and regulate their own activity correctly, i.e., to be an actor of their own activity, can be considered as a measure to prevent emotional burnout and may lead to effective personal development at an elderly age.

In Russian research papers, personal activity related to people of “the third age” is treated as a compensatory mechanism that can reduce or neutralize certain age-related losses. In particular, the research findings of Korsakova N.M. [24] suggest that a person’s activity is directed at forming effective ways to overcome changes in fulfilling higher mental functions. Besides, a person’s activity is considered as a potential for the future development of intellect [25,26], vitality and resilience [20,27], and self-regulation and self-identification [28]. 

Going through the crisis is accompanied by certain structural personality changes: in the professional direction (motivation, identification, values, and meanings), professional expertise, and professionally relevant personality features. 

Such structural personality changes are mostly specific to critical periods. Vygotsky L.S. argued that such changes are transitional, which means that “afterwards they are not preserved in the form they appeared in the critical period and are not included as a constituent into an integral structure of personality” [14]. Thus, the structural changes of an actor are temporary and they tend to disappear during stable periods of professional development.

There are two types of resolving the crisis: constructive and destructive [19]. Zeer E.F. added one more type to this list: a professionally-neutral type that does not exert any influence on professional activity [16]. The constructive type of solution includes significant changes in professional consciousness and activity, and the transition of a person to a higher level of development [15,19]. The constructive type is the only one that leads to the progressive development of a person. The destructive way of coping with a crisis may result in personality regress, an abrupt career switch, or departure from active professional activity. This is an irrational, passive, and inadequate reaction to a crisis or escape from it and allocates the responsibility of solving the contradictions to other people or circumstances. Distancing oneself from the signs of the crisis is possible in the form of family abandonment, career change, or transition to another age group. It indicates symptoms of obviating difficulties, inability to solve problems, and contradictions in a productive or persistent way.

The constructive way of dealing with the personality crisis imposes special requirements on the level of development of such parameters as activity, stability, integrity, values and attitudes, the level of subjective self-control, and professional orientation. These characteristics constitute a basis for a creative attitude to life and control over one’s own destiny.

Two strategies may be proposed in order to cope with professional development crises: a proactive strategy and a situational strategy. A proactive strategy is characterized by the manifestation of activity, purposeful behavior, and responsibility for the decisions and actions taken. The choice of the proactive strategy is a sign of a mature personality. While analyzing the features of a mature personality Anan’ev B.G. [27] put emphasis on the unity of internal situations, behavior tactics, and strategies with general ideas and worldviews. The situational strategy is characterized by a low level of personal integrity and the weakness and instability of inner cognitive, emotional, and volitional attitudes. Thus, situational ways of overcoming crises are caused by a lack of supraorganic structuredness and the conjugacy of individual moments of life; external orientation of the personality, asthenia and a low level of optimism, and the prevalence of bad forecasts of professional development. The situational strategy is dominated by mechanisms of psychological protection and adaptation to the surrounding reality. A proactive strategy is determined by contingency and connectedness of certain units (life attitudes) of an agent of activity in inner space and time, as well by an ability to build self-creation and self-construction, personality stability, value priorities, dynamic orientation of the personality, optimistic forecasting, personal responsibility, and focus on success and achievement. The proactive strategy implies an activity-based type of coping with the crises [15].

Overcoming profession loss crisis includes the reconsideration of a person’s ideas about themselves, finding new meanings in life, setting new goals and objectives, and adjusting self-perception and self-awareness. Psychological aid and support are very important at this age. One of the forms of such aid is social and psychological education. It will help to develop a culture of decent living standards, acquire personal hygiene, self-care, and household economy skills. The main thing is to start creating a timely psychological readiness for facing old age. 

In order to relieve the crisis effects, it would be justified to conduct courses on preparing for retirement, social and economic mutual aid, and to organize leisure clubs for pensioners.

The following research hypotheses were identified for this study:

—  Psychological characteristics of profession loss crisis include a decline in the level of professionalism, awareness of the need to reevaluate oneself a feeling of exhaustion of one’s capabilities, lowering one’s own assessment, lagging behind the times, tiredness, loss of a sense of novelty, and inner confusion;

—  Factors that predetermine profession loss crisis of the aged teachers are worsening of social and economic conditions, age-related psychophysiological changes, and dissatisfaction with individual needs;

—  In order to cope with profession loss crisis at a late age, teachers change workplaces and seek their colleagues’ help. It indicates the willingness of teachers to leave their profession.

## 2. Materials and Methods

A pilot research study of professional development crises was carried out during 2016–2018. 

### 2.1. Sample

Teachers of general education institutions of the Sverdlovsk region entering the study ranged in age from 50 to 60 years. The study sample included teachers of retirement and pre-retirement age, since the investigation of profession loss crisis will help develop effective technologies for psychological assistance and support. The sample of the study amounted to 191 people (20 men and 171 women). Currently, in educational institutions of the Sverdlovsk region, the percentage of teachers of pre-retirement age is 14% (8377 out of 57,658 persons) with 10.5% of men (878 persons) and 89.5% women (7499 persons). It corresponds to the gender distribution of the sample. All participants gave their informed consent before the beginning of the study. The study was conducted in accordance with the Helsinki Declaration, protocol No. 10 (17 October 2016) and was approved by the Scientific Council of the Institute of Social and Political Sciences of Ural Federal University.

### 2.2. Ethical Considerations

Research methods correspond to the research objectives. Participation in the study was voluntary. Participants in the study were competent people without physiological or mental disorders. All participants were informed about the aim and objectives of the study and provided informed consent to take part in it. The study participants were provided with confidentiality and anonymity; however, there was a clause in the informed consent on the possibility of anonymous use of individual statements as examples of the study. The research participants were entitled to withdraw from it at any time. The research procedure and the biographical questionnaire did not contain any unacceptable or insulting definitions and statements in reference to the participants. Their life during the study was not exposed to danger.

### 2.3. Method

The study was conducted using the psychobiographical method and the critical events method.

The biographical method contributes to analyzing a dynamic process of a person’s professional development at all stages: option, professional training, professional adaptation, primary and secondary professionalism, and mastery, as well as creating conditions for identifying critical periods in this process.

The psychobiographical method makes it possible to investigate professional crises in their development: to define the factors that determine crises and to identify structural changes the agent goes through when undergoing a crisis and ways to cope with it. Therefore, the application of the psychobiographical method seems to us the most effective for investigating professional development crises.

In order to study profession loss crisis, a formalized biographical questionnaire by Norkidze V.G. was used that was modified by Zeer E.F.

The methodology is a biography plan, which includes the following points:

Family background (parents, psychological climate in the family, siblings, wide family circle);

Social and economic conditions of life (economic characteristics of the family, characteristics of the place of residence);

Childhood history (date and place of birth, childhood experiences, past illnesses, childhood dreams of the future);

Professional development (professional intentions at preschool, primary school, adolescent, young ages; choice and implementation of professional intentions; professional expectations; restrictions on the choice of profession due to health conditions and abilities; impact of military service on professional intentions; entry into the profession; professional development crises; training);

Gender-related peculiarities of professional development (marriage effect on career; having children and their impact on professional biography; a desire that children would step into their parents’ professional path);

Vision of the future (plans for the next 2–3 years; longer-term plans; openness to change; clear vision of professional future in the current economic setting. 

### 2.4. Procedure 

The study was conducted jointly with the Ministry of Education and Youth Policy of the Sverdlovsk Region in secondary schools. After completing training on coping with professional destruction, the participants were invited to take part in the study. They were informed about its aim and procedure, provided written consent, and were given a form with a questionnaire according to which their psychobiographies were created. The scope of psychobiography was not limited. The time allotted for writing psychobiographies was 2 h. After writing a psychobiography, the participants were thanked for their contribution. The study did not provide for material reward. 

### 2.5. Data Processing 

Data processing was carried out using content analysis—a method for identifying and assessing specific characteristics of texts and other information items. 

The purpose of the content analysis was to identify the psychological characteristics of profession loss crisis for teachers. 

All statements that characterized crises or were related to their reasons, psychological changes in the structure of the agent and ways to overcome crises were considered to be the elements of the texts of psychobiographies. The research took both value judgments and simple descriptions into account.

Content-analysis categories are key notions that constitute a concept on which the research is based. A detailed description of categories in terms of the texts under study was constructed. Coding instructions were also compiled. The following categories were singled out: A—factors, which determine professional development crises, B—changes in substructures of the agent of activity, C—ways of overcoming crises. When drafting coding instructions within these categories, subcategories of the first- and second-order were singled out. The frequency of occurrence of subcategories was entered into matrices. Later a percentage interpretation was obtained in relation to a total number of selected categories of the analysis. Conclusions about the level of expression of the selected semantic units were made on the basis of the obtained results. 

In the process of data interpretation, a person’s active role in the retrospective assessment of their own professional experience was taken into account.

## 3. Results

Among the factors that initiate the crisis of profession loss (Table 1), a predominant role (97.9% and 2.1%) is played by objective factors. The most common factor is random events in life (44.3%). Their inclusion in the individual trajectory often exerts a powerful influence on all aspects of one’s life and it can trigger professional development crises. 

The influence of adverse circumstances when implementing professional plans (23.2%) and the deterioration of the social and economic situation in the country (21%) are reported to be the second most frequent factors. Responses in this category reflect the dependence of professional development crises on changes in social and economic conditions. It was caused by reaching the retirement age. This age is characterized by a number of changes. The first one to mention is psychophysiological involution, which means changes in the functioning of cognitive processes. The next factor refers to a change in the social situation: a person can be involved in professional activities, but other people consider him/her to be a pensioner and it may result in communication difficulties, as well as psychological stress, and may lead to complicating the process of professional activity. Retirement also leads to a deterioration in financial opportunities; it results in an unwillingness to retire and an attempt to stay at work at any cost and in any position. Many working retirees transfer to lower positions, which leads to a loss of status and causes psychological discomfort.

Analysis of the severity of changes in the structure of the actor’s activity in the process of profession loss crisis (Table 2) reflects a predominance of changes in the substructure of professional orientation (95.4%). Restructuring of the social and professional orientation is expressed in awareness of the discrepancy between the activities and expectations (67.5%). Substantial professional experience is a prerequisite for shaping the position of a super professional, but this often leads to a stagnation of professional development, limitations in professional activities, and loss of professional adequacy. The next in terms of severity is dissatisfaction with the activity performed (14%). This indicates that the crisis caused by adverse circumstances and random events affects the professional position of an individual.

The severity factor of the lack of prospects for a change in vocational education status amounts to 7% and reflects a person’s awareness of the need to change the previous situation. In this case, it is necessary to build up a professional perspective and think over ways to implement it. Thus, the continuation of professional activities can have different forms: mentoring and supervision or work as an organizer or chairman. In these options, a person does not leave their profession and their accumulated professional experience is socially useful [29]. 

However, the continuation of professional activity does not imply that a person remains involved in his previous profession and maintains the same position, as they can try a new professional role. But most likely it will be a lower-status profession in another field and it can produce constructive as well as destructive effects. If the professional activity itself seems to be valuable, it will help to achieve well-being and maintain contact with the world. If the employee values status or earnings, it will depress them and lead to his/her alienation. At the same time, dramatic changes in the usual professional activity can result in an awareness of one’s professional incompetence practically not being expressed (4.6%). 

The analysis of structural changes of the activity agent during profession loss crisis helped to determine that the most explicit changes are changes in social and professional orientation.

Overcoming profession loss crisis is more problematic and is accompanied by strong emotional concern. The strategies for overcoming profession loss crisis may be characterized in the following way (Table 3): The majority of registered responses reflect an initiative strategy of overcoming the crisis (83.7%). Responses that reflect a situational strategy amount to 16.3%. Coping with the crisis is often accompanied by “changing job” (30.2%). A person is forced to leave his former workplace but is not ready to be left jobless or to change their social role from an employee to a pensioner. This situation is controversial: if a person is obsessed with his social role as a representative of a particular profession it can lead to an inadequate self-image and consequently inadequate behavior. If a person accepts the role of a pensioner he adopts stereotypical forms of behavior and perceives himself as a burden and uselessness for society [30]. The most favorable constructive way out is the formation of a new positive identity rather than the adoption of a social role. On this basis, further meanings in life will be created [31]. “Seeking help from colleagues and administrators” is also considered to be a means of coping with the crisis (14%). Assistance from colleagues is perceived as luck, a happy coincidence: “I was lucky with my colleagues, everyone helped each other”, “I was lucky with my boss—I always received help and support”. Many teachers consider “retraining within the same profession” (11.6%) as a way out of the crisis, but with a lowering of status and decrease in job responsibilities, as well as changing profession (11%).

Achieving retirement age means inevitable retirement from work in most countries. In Russia, the situation is different—people can work after retirement. Thus, profession loss crisis is overcome by changing jobs, seeking help from colleagues and managers, retraining within the same profession or changing profession, increasing self-confidence and developing self-acceptance, supporting a person’s self-esteem and social significance. Teachers may stay at schools in other positions such as a methodologist, headteacher, etc.

It must be recognized that at a late age a person has the right and need to choose the meaning and purpose of their life, and, consequently, the possibility of progressive or regressive personality changes. In general, a free, albeit difficult, choice makes it possible to characterize this age as an age of development and potential possibilities giving a chance to resist decay. The final choice is determined by solving a task to define the meaning of one’s remaining lifetime. In accordance with this choice and, consequently, adaptation strategy, major activity may be aimed either at preserving one’s personality (maintaining and developing his/her social ties), or at isolating, individualizing, and his/her “surviving” with simultaneous deterioration in physical, physiological, and psychophysiological functions.

Both variants of aging obey the laws of adaptation, but provide a different quality of life and even its duration. Much of the literature has highlighted the second variant of aging, in which age-related changes manifest themselves in a qualitatively peculiar reorganization of the organism, preserving specific adaptive functions against the background of their decline. This adaptation strategy involves a gradual restructuring of the main vital processes and the overall structure of the regulation of functions in order to ensure the safety of the individual and maintain or increase life expectancy. This adaptation strategy involves a transformation of the person’s “open” system into a “closed” one. An alternative adaptation strategy is observed when an elderly person seeks to preserve oneself as an individual; it is connected with maintaining or developing his/her ties with society. In this case, the structuring and transfer of experience can be considered a leading activity in old age. In other words, a positive evolution at an old age is possible if an elderly person finds the opportunity to use the accumulated experience in a socially meaningful context and invest a part of their individuality and soul into it.

## 4. Discussion 

The research findings clearly indicate that the retirement crisis is accompanied by social and professional changes. Their effects can be softened by special training that prepares employees for retirement. Previous research has shown that such programs enhance one’s dedication to working, improve satisfaction through an awareness of contributing to the common good, and decrease stress levels [32,33].

The results of this study suggest that finding meaning in life in the nonprofessional sphere was not the main way to overcome profession loss crisis. However, community service may become a way to find new meanings in life (not related to one’s professional sphere) after retirement. As Pundt, Wöhrmann, and Deller and Shultz underlined, volunteering allows retirees to meet needs for achievement, acceptance, autonomy, social ties, and sharing experiences with the younger generation [34]. 

Positive evolution in later life is possible if a person finds the opportunity to apply their own experience in a socially significant activity. Therefore, past experience of mentoring contributes to satisfying a need to share experience and develop ego-identity [35]. 

Survey results reveal that very often retired people do not consider their loved ones as a resource. A habit of living at work limits the resource possibilities in overcoming this crisis. The resources, in this case, are the presence of life meanings, psychological and somatic health conditions, and self-evaluation, as well as cognitive and intellectual abilities. With excessive professional activity, all meanings in life that are not related to work are lost, health deteriorates due to excessive overload, and cognitive processes lose their flexibility.

A positive way of coping with this crisis may be achieved by psychological support for people of pre-retirement and retirement age. This process involves various forms of advisory work on adopting age-related changes, professional self-determination, and training of cognitive skills to maintain their tone. 

Many researchers have underlined the need to study and develop technologies of “active aging”, aimed at improving the quality of life of young people by creating opportunities to maintain their health and satisfy security and affiliation needs. For example, in a study conducted in Brazil, a sample of 1006 elderly people aged 60 and over was selected to investigate a relationship between mental health and active aging [36]. Active aging was evaluated by the following parameters: professional activity, physical activity, communication with friends, and reading. The study proved, with approximately equal social, economic, demographics, and medical backgrounds, those who continue to work, engage in physical labor, read, or communicate with friends are less subject to depression than people who are not engaged in such activities. This gives cause to conclude that active aging is an effective strategy for maintaining and strengthening mental health.

The need to develop active aging technologies is also associated with the fact that due to a rise in life duration and drop in the birth rate in Europe the share of the working-age population from 55 to 64 years old will increase by 16% in the coming decades, whereas the share of the working-age population of other ages will decrease. At the same time, the occupation level of employees aged 55 to 64 years is less than 50%. It means that more than half leave work before achieving retirement age. Mucci [37] emphasizes that this trend is destructive for European economies, and, therefore, it is necessary to extend the professional life of elderly people through active aging technologies. The working environment is an ideal platform for promoting active aging, for supporting the psychophysical abilities of employees and preserving their ability to work, and for developing cooperation between generations.

Finnish researchers recently provided an example of an age management model. The multi-level model is based on a comprehensive study of the work capacity of older people. According to Ilmarinen [38], this model involves continuous interaction between employers and employees and the synergy of their actions. Employees should be responsible for their health and employers should promote a healthy lifestyle, monitor professional hygiene factors, minimize health risks, and organize work in compliance with age-specific features: a flexible schedule, less physical activity, etc. Employees should be interested in building their own competencies, the organization should provide opportunities for them to acquire new skills. Learning, as emphasized by Ilmarinen, is a critical success factor for active aging. It is also necessary to take into account and use the strengths of older employees: strategic thinking, insight, wisdom, self-control, dedication, and loyalty to the organization. They would provide grounds for distributing responsibilities between younger and older employees. An essential factor to ensure the efficiency of the elderly is a suitable dialog between employers and employees: people should feel that they can trust and respect the employer. They need support and feedback on the results of their activities and should receive fair treatment without age discrimination. A key role is also played by the immediate social environment, i.e., family and friends, and intrapersonal characteristics, i.e., motivation, value systems, and the ability to transmit experiences to younger generations.

According to the data provided by Ilmarinen, the concept of active aging and an increase in the length of professional can prevent disease in older people and helps to maintain their mental health, physical and cognitive abilities, and contributes to the sustainability and progressive development of society [38]. 

## 5. Conclusions

Thus, profession loss crisis is characteristic of the last stage of professional development.

1. Due to random events (44.4%), unfavorable circumstances (23.2%), or worsening socio-economic conditions (21%), this crisis disrupts the path of professional development. Objective factors (97.9% and 2.1%) play a predominant role. Retirement is a very important stress factor for a person as this entails a deterioration in financial opportunities and an abrupt change in social status. People of pre-retirement and retirement age experience anxiety, mood fluctuations, and proneness to conflict in a professional sphere. It can be illustrated by such statements as: “there was a conflict with the administration”, “there is no stability”, “there is enough money only for food”, among others. All these statements reflect objective factors in the development of profession loss crisis.
2. Restructuring of the person’s activity that accompanies the process of profession loss crisis reflects the predominance of changes in the substructure of the professional orientation (95.4%). Awareness of the discrepancy between professional activity and expectations is characteristic of this crisis (67.5%). Rich professional experience gives rise to higher expectations in terms of a professional career, but the psychophysiological and organizational abilities at this age are limited in comparison to the earlier periods of one’s professional development. This can result in stagnation, limited activity, and a loss of professional adequacy. It can be illustrated by the following statements: “a lot of disappointment, plans were not fulfilled, expectations were not met”, “lost oneself, one’s meaning in life”. Of the respondents, 14 percent indicate that their professional duties are considered a burden. It leads to a change in the professional position of the individual when “work no longer gives pleasure”.
3. Coping with profession loss crisis is more problematic and is accompanied by strong emotional experiences. Teachers’ psychobiographies reveal that the most common ways out from these crises are changing jobs (30.2%), seeking help from colleagues and superiors (14%), and retraining within the same profession (11.6%). The initiative strategy of overcoming profession loss crisis helps a person remain active, resist psychological and psychophysiological age-related changes, and enhance the quality of later life. The most favorable and effective way out of profession loss crisis is not the adoption of a social role but rather a formation of a new positive identity: on the basis of which further meanings in life will be created.
4. Psychological follow-up of the people of the pre-retirement and retirement age can contribute to a successful resolution of profession loss crisis. It will be implemented in Russia as a part of the national project “Demography” and a subproject “Senior Generation”. The project objectives include rendering social services for people of old age, the stimulation of active longevity, development of a geriatric care system, and professional retraining within working professions (carpenter, baker, welder). However, the technological revolution 4.0 is leading to profession loss crisis among lawyers, economists, engineers, and other highly qualified specialists. The results of our study, conducted on a group of teachers, can constitute the basis for a system of psychological support for people of high social status who undergo profession loss crisis. It is also planned to develop various forms of consulting work on the adoption of the age-related changes and professional self-determination, and to conduct training to enhance cognitive skills. The result of the psychological support should be an increase in the quality of life in late adulthood, self-realization in all spheres where profession loss crisis should be considered as “one more opportunity for a person’s development”.

## 6. Limitations 

The findings of this study have to be seen in light of some limitations. First of all, this concerns the specifics of the sample as it consists of the representatives of one professional group (teachers in the sphere of general education) with an overwhelming prevalence of women. It reduces the possibility to generalize the conclusions. Therefore, the results obtained from female feedback cannot be fully applicable to male pre-retirement and retirement age groups. The study group also consisted of the representatives of general education teachers, which reduces the feasibility of its results for representatives of other professional groups. A natural progression of this work is to analyze profession loss crisis among engineers and economists, since their job content is different and a comparative study would be useful.

The psychobigraphic method did not take into account data concerning the quality of a person’s professional and personal biography: whether they were successful or not. Further studies should focus on factors, peculiarities, and effective ways to cope with the crisis in the context of the entire professional biography.

The last limitation is that the results are characteristic of a Russian sample. Social, economic, and cultural peculiarities of the retirement process in Russia do prevent the application of the results of the study to representatives of other countries. This shortcoming can be overcome in the future by conducting cross-cultural research.

## Figures and Tables

**Table 1:** Factors of profession loss crisis.

Loss of Profession Crisis	%	Responses
Objective 97.9%		
A1—change in the social situation of development	4.3	«It’s difficult in a new situation, no contact with students», «A difficult group that initiates an open conflict with teachers», «Conflict with management»
A2—age-related psychophysiological changes	5	«Because of bad eyesight I wasn’t allowed to a recruitment competition», «Poor health», «Tired legs from long standing», «Got tired faster than before»
A3—improvement or deterioration of social and economic conditions	21	«There was a lack of stability», «Have money only for food, no money even to buy books»
A4—random events	44.4	«I realized that activity results depend not only on me»
A5—adverse circumstances	23.2	«Everything collapsed at once», «Son’s illness and disability», «Divorce»
Subjective 2.1%		
A6—unsatisfied needs of an individual	2.1	«No job satisfaction», «Frustration from work», «Treating me as a third-rate person»

**Table 2:** Changes in the structure of the actor’s activity during profession loss crisis.

Changes in the Structure of the Actor’s Activity During Profession Loss Crisis	%	Responses
Changes in the sub-structure of professional orientation 95.4%		
B1—loss of meaning of educational and professional activities;	3.9	«Lack of interest to this job», «Didn’t see a point of doing this», «My job turned out to be useless»
B2—dissatisfaction with the process of performing the activity;	14	«I realized that work must bring satisfaction, be useful to someone», «I wanted to try myself in a new capacity, because the work was no longer satisfying»
B3—anxiety over a lack of opportunities for self-expression and self-actualization	3	«No opportunity for self-expression», «I wanted more creative work»
B4—lack of prospects for changing social and professional status	7	«I wanted professional growth», «Desire for higher social status»
B5—anxiety over a mismatch of professional activity with expectations	67.5	«Lots of frustrations, unrealized plans, expectations are not met», «Losing touch with reality», «Hasn’t achieved anything», «Lost myself, the core of life»
Changes in the sub-structure of social and professional competence 4.6%		
B6—awareness of one’s own incompetence	4.6	«There was a lack of knowledge and competence; I had to prepare a lot, learn anew, start from scratch», «Lack of knowledge», «Lack of expertise»

**Table 3:** Strategies for coping with crisis.

Strategies for Coping with Crisis	%	Responses
Initiative Strategy 83.7%		
C1—training and development	5	«Studies and training», «Entered training and development courses», «Continual self-education», «Training and development within the main profession»
C2—change of job	30.2	«workplace change»
C3—revision of meaning in life and opportunities, drafting new scenarios of professional life;	6.9	«Ability to find joy in one’s place», «The main thing is that children ask for help and advice; this is professional happiness», «Passion for the profession, love for children»
C4—seeking help from colleagues and administrators	14	«Got assistance from colleagues», «There was a good mutually-supportive team»
C5—retraining within the same profession	11.6	«Retraining within my profession», «I went to teach a profession, not to work»
C6—return to the same workplace and position	5	«I accepted a new position but after working for while returned to my former workplace with increased professional level»
C7—change of profession	11	«Quitted from work», «Changed profession»
Situational Strategy 16.3%		
C8—finding meaning in life in non-professional sphere	4.3	«Got fascinated by social work»
C9—coping with the crisis due to pressure from parents, friends, and colleagues	2.5	«Was invited to the work by a headmaster»
C10—a compromise resolution of a problem that does not eliminate contradiction, but relieves stress	3.3	«I continue to do this work, since there is nowhere else to work in the village», «Left the institute»
C11—gradual reconciliation with the activity	3.2	«I was a housewife, a feeling of loneliness made me go to work again and I enjoy it»
C12—a random way out which turned out to be successful	3	«Got adjusted like anyone else», «got lost in daily routine», «Got used to it gradually»
